# The critical role of inflammation in osteoporosis prediction unveiled by a machine learning framework integrating multi-source data

**DOI:** 10.3389/fphys.2025.1729997

**Published:** 2025-12-10

**Authors:** Bo Liu, Hongli Chang, Peipei Li, Hongguang Chang, Xuenan Wang, Wubing He

**Affiliations:** 1 Shengli Clinical Medical College of Fujian Medical University, Fuzhou, China; 2 Institute of Brain Science and Brain-Inspired Research, Shandong First Medical University (Shandong Academy of Medical Sciences), Jinan, China; 3 School of Continuing Education, Shandong First Medical University (Shandong Academy of Medical Sciences), Jinan, China; 4 Department of Emergency Surgery, Fujian Provincial Hospital, Fuzhou, China; 5 Fuzhou University Affiliated Provincial Hospital, Fuzhou, China

**Keywords:** osteoporosis, machine learning, inflammatory biomarkers, predictive models, multicenter study

## Abstract

**Objective:**

Osteoporosis poses a major global public health challenge. The limitations of current diagnostic methods, primarily diagnostic delays in bone density testing, are compounded by the insufficient exploration of inflammatory factors in predictive models for the disease’s pathogenesis. This study aims to leverage multi-source data and machine learning to explore the value of inflammatory markers for osteoporosis prediction, establishing a high-precision model for early screening and precise prevention.

**Methods:**

A multi-center, multi-level research design was employed, integrating four independent datasets: the National Health and Nutrition Examination Survey (NHANES) database (12,988 adult women), a Chinese postmenopausal women specialized cohort (CPW-BMI) (312 participants), the Osteoporosis Phenotype Validation Cohort (OP-VC) (60 participants), and animal experimental data (40 C57BL/6J mice). A predictive indicator system comprising 22 clinical features and inflammatory markers was constructed. Various machine learning algorithms (including RUSBoosted Trees, Bagged Trees, Support Vector Machines, Gaussian Process Regression, etc.) were used to establish classification and regression prediction models, and model performance was evaluated through rigorous five-fold cross-validation and external validation.

**Results:**

Machine learning models based on inflammatory markers exhibited excellent predictive performance across different bone sites. At the femoral neck, the RUSBoosted Trees model achieved an Area Under the Receiver Operating Characteristic Curve (AUC) of 0.9643 and an accuracy of 90.55%; at the lumbar spine, the Efficient Logistic Regression model achieved an AUC of 0.9685 and an accuracy of 91.79%. External validation demonstrated good generalization ability: in the Chinese population cohort, the Fine Gaussian Support Vector Machine model had a prediction error (Root Mean Square Error, RMSE) of 0.681; in the clinical cohort, serum levels of Interleukin-6 (IL-6), Tumor Necrosis Factor-alpha (TNF-α), and Interleukin-1 beta (IL-1β) were significantly elevated in the osteoporosis group; in animal experiments, a Linear Discriminant Analysis model based on three core inflammatory factors achieved 97.5% accuracy (AUC = 0.9574). These results confirm the value of inflammatory markers in osteoporosis risk assessment.

**Conclusion:**

Using inflammation markers and machine learning, we created accurate models to predict osteoporosis. This work confirms inflammation’s key role in the disease, providing new insights for early detection and targeted intervention.

## 1 Introduction

Osteoporosis, a systemic skeletal disorder characterized by diminished bone mass and microarchitectural deterioration of bone tissue, leads to increased bone fragility and a consequent elevated risk of fractures (Srivastava and Deal, 2002; Lane et al., 2000; Lane, 2006). It has emerged as a critical global public health challenge, severely impacting the quality of life, particularly among postmenopausal women (Black and Rosen, 2016; Brown, 2021). According to the International Osteoporosis Foundation, approximately 250 million people worldwide are affected, with the prevalence soaring to 30%–50% in women over 50 (Coughlan and Dockery, 2014; Subarajan et al., 2024). The accelerating aging population in Asia, especially in China, has further intensified the challenges in osteoporosis management (Chandran et al., 2023). Osteoporotic fractures, the most severe complication, now occur at an annual incidence rate surpassing the combined incidence of myocardial infarction, stroke, and breast cancer (Liu et al., 2025). Notably, hip fractures are associated with a 1-year mortality rate of 20%–24%, imposing a substantial economic burden on families and society (Cummings and Melton, 2002; Marks et al., 2003).

In current clinical practice, the diagnosis of osteoporosis primarily relies on Bone Mineral Density (BMD) measured by Dual-energy X-ray Absorptiometry (DXA) (Lane, 2006). However, this conventional diagnostic paradigm has significant limitations. Firstly, BMD testing is often performed only after patients present with clinical symptoms or sustain fragility fractures, representing a “post-diagnosis” model that misses the optimal window for early intervention (Howe et al., 2011). Secondly, BMD accounts for only about 70% of bone strength, failing to fully capture other crucial determinants of bone quality, such as bone microarchitecture, bone turnover rate, and bone material properties (Seeman and Delmas, 2006; Boskey and Imbert, 2017). Furthermore, traditional diagnostic criteria have not adequately incorporated the role of chronic low-grade inflammation, a key player in the pathogenesis of osteoporosis (Iantomasi et al., 2023; Wu et al., 2021; Luo et al., 2025; Chotiyarnwong and McCloskey, 2020).

Recent advances in molecular biology and immunology have increasingly highlighted the pivotal role of inflammatory cytokine networks in the development and progression of osteoporosis (Saxena et al., 2021; Fischer and Haffner-Luntzer, 2022; Eastell and Szulc, 2017). Under conditions of estrogen deficiency, the monocyte-macrophage system becomes activated, leading to a significant upregulation of pro-inflammatory cytokines such as (IL-6), TNF-α, andIL-1β (Joyce et al., 1997). These inflammatory mediators regulate bone metabolism through multiple pathways: they directly activate the Receptor Activator of Nuclear Factor Kappa-B Ligand (RANKL)/Receptor Activator of Nuclear Factor Kappa-B (RANK)/Osteoprotegerin (OPG) signaling path way to promote the differentiation and maturation of osteoclast precursors; they stimulate bone marrow stromal cells to secrete more RANKL while inhibiting OPG production, further exacerbating osteoclast activity (Walsh and Choi, 2014); and they suppress osteoblast differentiation via the Wingless (Wnt)/β-catenin signaling pathway. The resultant imbalance between bone resorption and formation accelerates bone loss (Hu et al., 2024).

In the realm of osteoporosis risk prediction and early screening, existing tools like the fracture risk assessment tool (FRAX) algorithm primarily rely on a limited set of clinical risk factors (Hsu et al., 2020; Davis et al., 2020). They lack integration of systemic inflammatory biomarkers and exhibit significant variability in predictive performance across different ethnic and geographical populations. The advent of the medical big data era and rapid developments in artificial intelligence offer a new technological pathway for early prediction and precise diagnosis of osteoporosis using machine learning. Machine learning algorithms can uncover complex patterns from high-dimensional, multi-source medical data to construct non-linear predictive models, potentially overcoming the limitations of traditional statistical methods (Sparks et al., 2024; Lane et al., 2025; Liu et al., 2024; Hong et al., 2024).

Nonetheless, current machine learning-based research on osteoporosis prediction faces several challenges: most studies depend on single data sources, limiting model generalizability (Deo, 2015); there is a lack of systematic integration and evaluation of inflammatory markers; model validation often stops at internal checks, lacking multi-center, multi-level external validation; and crucially, the connection between predictive models and underlying biological mechanisms remains largely unexplored, hindering clinical translation and application (Eraslan et al., 2019). Specifically, many models have failed to systematically integrate readily accessible systemic inflammatory markers into the predictive framework, and the models’ generalizability along with their potential biological interpretability are often insufficient, thereby limiting their clinical translational potential.

To address these gaps, our study employs a multi-level, multi-dimensional research strategy aimed at constructing and validating a machine learning prediction framework for osteoporosis based on inflammatory biomarkers. First, we utilized the nationally representative NHANES database as our development set to systematically evaluate the predictive value of 22 feature variables, including composite inflammatory indices, and performed detailed performance comparisons across different skeletal sites (femoral neck, lumbar spine, and femoral trochanter). Second, to ensure model generalizability and clinical applicability, we designed three independent validation tiers: CPW-BMI dataset; OP-VC dataset; and finally, using an Ovariectomized (OVX) mouse model to experimentally validate the critical role of inflammatory factors in bone metabolism and build a disease status identification model based on these factors.

The innovativeness of this study is multi-faceted: it is the first to systematically incorporate composite inflammatory indicators derivable from routine blood tests—Neutrophil-to-Lymphocyte Ratio (NLR), Platelet-to-Lymphocyte Ratio (PLR), and Platelet-to-Neutrophil Ratio (PNR)—into an osteoporosis prediction model; it adopts a multi-level validation strategy from macro-population data to micro-animal experiments, building a comprehensive evidence chain; and it not only focuses on predictive performance but also delves into the intrinsic link between predictors and the pathogenesis of osteoporosis, providing a solid biological rationale for the model. Furthermore, we innovatively applied advanced machine learning algorithms like Gaussian Process Regression and Ensemble Learning to predict continuous BMD values, offering a more refined risk assessment tool for clinical practice.

Through this systematic investigation, we aim to establish an accurate, robust, and interpretable early prediction model for osteoporosis, providing clinicians with an effective screening and diagnostic tool for early identification and precise intervention. Simultaneously, this study will offer new experimental evidence to deepen the understanding of inflammation’s role in osteoporosis pathogenesis, laying a theoretical foundation for developing novel prevention and treatment strategies targeting inflammatory pathways. From a broader perspective, our findings hold potential for translation into clinical practice, improving patient outcomes, reducing the incidence of osteoporotic fractures, and generating significant social and health economic benefits. Ultimately, we anticipate that this interdisciplinary research approach will open new avenues for preventing and managing osteoporosis, advancing the application of precision medicine in the field of skeletal health.

## 2 Materials and methods

### 2.1 Data sources and description

#### 2.1.1 NHANES dataset (primary training and internal validation set)

All participant information was extracted from the National Health and Nutrition Examination Survey (NHANES), which is designed to assess the nutritional and health status of the general US population based on a cross-sectional design. NHANES is part of the US Centers for Disease Control and Prevention and is updated every 2 years. NHANES participants signed informed consent forms before the implementation of the NHANES protocol approved by the NCHS Research Ethics Review Board (Prevention NCfHSCfDCa, 2024).

We extracted data from NHANES 1999–2020 (including cycles 1999–2000, 2001–2002, 2017–2020). Samples with missing values and outliers were excluded. All adult women aged ≥18 years with complete data were included in the final analysis.

#### 2.1.2 CPW-BMI dataset (external validation set I)

This dataset was sourced from the Figshare scientific data sharing platform (DOI: 10.6084/m9.figshare.25526551.v2). It employed a single-center cross-sectional study design and was collected from a tertiary Grade A hospital in China between January 2021 and June 2023. The study included 312 naturally postmenopausal women aged 45–80 years (mean age 62.3 ± 8.7 years). All participants met the following inclusion criteria: 1) natural menopause for ≥1 year; 2) age ≥45 years; 3) clear consciousness and ability to independently complete questionnaires and physical examinations. Strict exclusion criteria included: 1) secondary osteoporosis; 2) severe hepatic or renal insufficiency (estimated glomerular filtration rate, eGFR <30 mL/min/1.73 m^2^); 3) history of any malignancy; 4) active phase of autoimmune diseases; 5) recent use (within 6 months) of medications known to affect bone metabolism (e.g., bisphosphonates, teriparatide, glucocorticoids, etc.).

#### 2.1.3 OP-VC dataset (external validation set II)

This study was a prospective investigation approved by the Biomedical Ethics Committee of the 900th Hospital of the Joint Logistics Support Force (Approval No.: 2025–079). Sixty naturally postmenopausal women aged 50–75 years were strictly recruited. Through a rigorous matching procedure (ensuring no statistically significant differences in baseline characteristics like age and Body Mass Index (BMI), P > 0.05), participants were divided into three groups: osteoporosis group (n = 20, femoral neck or lumbar spine BMD T-score ≤−2.5 SD), osteopenia group (n = 20, −2.5 ≤ T-score <−1.0 SD), and healthy control group (n = 20, T-score ≥−1.0 SD). Exclusion criteria included secondary osteoporosis, severe hepatic or renal insufficiency, history of malignancy, long-term use of bone metabolism-affecting drugs, and recent fracture (within 3 months).

#### 2.1.4 Animal Experiment Data (mechanistic exploration set)

Forty 12-week-old healthy female C57BL/6J mice (supplied by Beijing Vital River Laboratory Animal Technology Co., Ltd.), weighing 25–30 g, were used. All mice were housed in a specific pathogen-free environment under strictly controlled conditions (temperature 22 °C ± 2 °C, humidity 50% ± 10%, 12-h light/12-h dark cycle) with free access to food and water. The animal experimental protocol was approved by the Experimental Animal Ethics Committee of Shandong First Medical University (Approval No.: W202507040809).

### 2.2 Data collection

NHANES: As shown in Figure 2A, all datasets comprise 22 clinical examination features, including age, menopausal status, body mass index (BMI), corticosteroid use, diabetes status, smoking history, alcohol consumption, white blood cell count (WBC), lymphocyte percentage (LYMPH%), monocyte percentage (MONO%), segmented neutrophil percentage (NEUT%), eosinophil percentage (EO%), basophil percentage (BASO%), lymphocyte count (LYMPH, in 1,000 cells/μL), monocyte count (MONO), neutrophil count (NEUT), eosinophil count (EO), basophil count (BASO), platelet count (PLT), neutrophil-to-lymphocyte ratio (NLR), platelet-to-neutrophil ratio (PNR), and platelet-to-lymphocyte ratio (PLR). Detailed statistical information for the three datasets collected is presented in Table 1.

**TABLE 1:** Database information statistics.

Dataset		Femoral neck dataset		Spine dataset		Trochanter dataset	
Sum		n = 12,988		n = 10,787		n = 12,988	
Class		Normal group n = 9,797	Osteopenia group n = 3,191	Normal group n = 7,640	Osteopenia group n = 3,147	Normal group n = 6,255	Osteopenia group n = 6,733
Menopause		n = 1,500	n = 1,480	n = 1,126	n = 1,196	n = 684	n = 2,296
BMI(kg/m^2^) (mean ± std)		29.29 ± 5.92	26.16 ± 5.13	29.26 ± 6.18	26.76 ± 5.57	30.11 ± 5.93	27.05 ± 5.47
Age (year)		44.24 ± 13.56	54.00 ± 11.36	43.13 ± 13.12	26.76 ± 5.57	44.59 ± 13.54	48.55 ± 13.60
IF mean ± std	WBC	7.22 ± 2.29	7.00 ± 2.05	7.26 ± 2.46	7.05 ± 2.03	7.19 ± 2.40	7.13 ± 2.08
	LYMPH%	31.34 ± 8.23	30.98 ± 8.41	31.28 ± 8.28	31.49 ± 8.13	31.19 ± 8.25	31.30 ± 8.29
	MONO%	7.86 ± 2.30	7.86 ± 2.16	7.80 ± 2.31	7.76 ± 2.15	7.98 ± 2.34	7.75 ± 2.20
	NEUT%	57.25 ± 9.30	57.64 ± 9.37	57.41 ± 9.35	57.21 ± 9.17	57.23 ± 9.29	57.45 ± 9.34
	EO%	2.89 ± 2.17	2.84 ± 2.20	2.86 ± 2.13	2.85 ± 2.24	2.94 ± 2.13	2.82 ± 2.22
	BASO%	0.70 ± 0.50	0.74 ± 0.44	0.70 ± 0.50	0.72 ± 0.47	0.70 ± 0.49	0.72 ± 0.48
	LYMPH	2.20 ± 0.70	2.11 ± 0.70	2.21 ± 0.88	2.16 ± 0.68	2.18 ± 0.71	2.17 ± 0.69
	MONO	0.55 ± 0.18	0.54 ± 0.18	0.55 ± 0.19	0.53 ± 0.17	0.56 ± 0.18	0.57 ± 0.18
	NEUT	4.22 ± 1.84	4.11 ± 1.64	4.25 ± 1.94	4.11 ± 1.61	4.20 ± 1.94	4.18 ± 1.66
	EO	0.21 ± 0.20	0.20 ± 0.18	0.20 ± 0.20	0.20 ± 0.18	0.21 ± 0.20	0.20 ± 0.18
	BASO	0.04 ± 0.07	0.04 ± 0.06	0.04 ± 0.08	0.04 ± 0.06	0.04 ± 0.08	0.04 ± 0.06
	PLT	256.07 ± 67.14	251.47 ± 65.96	259.02 ± 67.23	256.65 ± 66.67	252.83 ± 65.56	256.89 ± 68.04
	NLR	2.06 ± 1.03	2.13 ± 1.25	2.07 ± 1.09	2.04 ± 1.03	2.07 ± 1.06	2.08 ± 1.12
	PNR	1,111.68 ± 1,022.13	1,061.50 ± 596.11	1,136.75 ± 1,120.81	1,082.63 ± 605.69	1,096.64 ± 1,180.41	1,101.86 ± 628.68
	PLR	126.17 ± 47.69	130.39 ± 51.57	127.70 ± 48.85	128.43 ± 48.52	125.68 ± 47.55	128.63 ± 49.72
Other index	Steroid	n = 16	n = 5	n = 11	n = 6	n = 11	n = 10
	Diabetes	n = 1,019	n = 392	n = 750	n = 304	n = 691	n = 720
	Smoking	n = 4,519	n = 1,513	n = 3,414	n = 1,394	n = 2,900	n = 3,132
	Drink	n = 7,651	n = 2,324	n = 5,915	n = 2,220	n = 5,016	n = 4,959

CPW-BMI Dataset Cohorts and OP-VC Dataset Cohort: All participants provided fasting venous blood samples collected in the morning. Blood samples were immediately placed at 4 °C, centrifuged at 3,000 rpm for 15 min within 2 h to carefully separate serum, aliquoted, and stored at −80 °C until analysis. Detailed statistical information is presented in Supplementary Tables S1, S2.

Animal Experiments: After 1 week of acclimatization, mice were randomly assigned to sham-operated (Sham, n = 20) or ovariectomized (OVX, n = 20) groups. All surgical procedures were performed under inhalation anesthesia with isoflurane (4% for induction and 2% for maintenance, delivered via a nose cone). Bilateral ovaries were completely removed aseptically in the OVX group, while the Sham group had an equivalent mass of peri-ovarian adipose tissue removed. Mice were housed individually post-operation and received daily intraperitoneal penicillin injections (50,000 units/mouse) for 3 days to prevent infection. At 12 weeks post-surgery, the experiment was terminated. Mice were transferred to an isoflurane anesthesia induction chamber (4%). After loss of consciousness, they were maintained under anesthesia via a nose cone (2%). The depth of surgical anesthesia was confirmed by the absence of a withdrawal reflex to a toe pinch, after which terminal blood collection was performed via the retro-orbital plexus. Following blood collection, samples were centrifuged at 4 °C and 15,000 × g for 4 min to isolate plasma. Immediately thereafter, mice were placed in a sealed chamber pre-filled with a high concentration of isoflurane and exposed continuously for at least 1 min after cessation of respiration, followed by an additional 1-min period to ensure death. Bilateral femurs were then carefully dissected for subsequent analysis.

### 2.3 BMD measurement

Human Cohorts: All participants (including those in the final analysis) underwent dual-energy X-ray absorptiometry (DXA) to assess bone mineral density (BMD). This test was performed by certified radiology technicians using a Hologic QDR-4500A fan-beam densitometer (Hologic Inc., Bedford, MA, USA). All DXA scan data were analyzed using Hologic APEX software (version 4.0). Detailed information is available on the NHANES website. Based on BMD at the femoral neck, femoral trochanter, and spine, participants were categorized into two groups: normal, and osteopenic or osteoporotic. Osteopenia and osteoporosis were defined as previously described, using the average BMD of 20–29-year-old white women as the reference. Any participant with a BMD T-score ≤−1.0 was classified as having osteopenia or osteoporosis (collectively termed ‘low BMD’), while those with a T-score >−1.0 were considered normal. In the CPW-BMI and clinical validation cohorts, DXA (GE Lunar or Hologic Discovery series densitometers) was used to measure BMD T-scores at the Total femur, following strict operational protocols and daily quality control (Zeng et al., 2019; Binkley et al., 2005; Looker et al., 1998; Looker et al., 2012).

Animal Experiments: The femur of each mouse was fixed in 4% paraformaldehyde for 48 h and then scanned using a Skyscan 1,276 high-resolution micro-CT imaging system. Scanning parameters were: X-ray voltage 55 kV, current 200 μA, integration time 300 m, and scan resolution 10 μm. Starting from 0.5 mm below the distal femoral growth plate, 300 consecutive image slices were acquired towards the diaphysis. Images were reconstructed using NRecon software, aligned using DataViewer, and subjected to 3D reconstruction and quantitative morphometric analysis using CTAn software. Evaluated parameters included: trabecular number (Tb.N), trabecular thickness (Tb.Th), trabecular separation (Tb.Sp), and structural model index (SMI).

### 2.4 Covariates

NHANES Covariates: The following variables were included as covariates in statistical models: age, menopausal status, smoking status, alcohol consumption, BMI, history of diabetes, and steroid use. Menopausal status was defined based on the reproductive health questionnaire: answering ‘No’ to ‘Have you had at least one menstrual period in the past 12 months?’ followed by indicating ‘Hysterectomy’ or ‘Menopause/change of life’ as the reason. Smoking status was defined as having smoked at least 100 cigarettes in one’s lifetime. Alcohol consumption status was defined as consuming 12 or fewer alcoholic drinks of any type per year. BMI was calculated from measured height and weight (kg/m^2^). Diabetes and steroid use were determined based on self-reported diagnosis or medication use.

Clinical Validation Cohort and Animal Experiments: This study focused on core inflammatory cytokines. Serum (human) or plasma (mouse) levels of IL-1β, IL-6, and TNF-α were measured using commercial ELISA kits (Shanghai Enzyme Link, Mlbio), strictly following the manufacturer’s instructions. All assays were performed in the same batch under identical experimental conditions, with duplicate wells, and the intra-assay coefficient of variation was controlled within 5%.

### 2.5 Machine learning analysis

**FIGURE 1:**
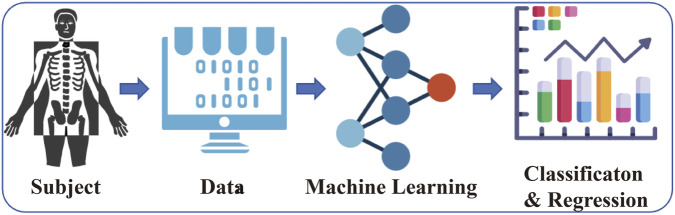
Ml for prediction of bone mineral density.

We employed various ML methods to classify and predict BMD, aiming to enhance the early detection and risk assessment of osteopenia and osteoporosis by analyzing individuals’ physical examination data and inflammatory markers. The specific methods for classification and regression tasks are detailed below, as shown in Figure 1A.

#### 2.5.1 Analysis strategy by dataset

NHANES: Two primary prediction tasks were conducted based on this dataset. 1) Classification Task: Convert BMD T-scores into binary labels – “Normal Bone Mass” (label = 0, T-score >−1.0) vs. “Low BMD” (label = 1, T-score ≤−1.0, encompassing osteopenia and osteoporosis) – aiming to identify individuals at risk for osteoporosis. 2) Regression Task: Use continuous BMD values (g/cm^2^) directly as the prediction target, aiming for a more refined assessment of bone density levels. Models were trained and evaluated separately for the femoral neck, spine, and femoral trochanter to investigate site-specific predictions.

CPW-BMI Dataset: The primary purpose was external validation. The best-performing machine learning models trained on the NHANES data were directly tested on this Chinese population cohort to evaluate their cross-ethnic generalizability and predictive accuracy.

OP-VC Dataset: This cohort served two main purposes: 1) To validate the association between inflammation and osteoporosis severity by comparing inflammatory cytokine levels among the three groups from a clinical chemistry perspective; 2) To retrain and validate machine learning models using the prospective data from this cohort, examining the predictive power of inflammatory factors for femoral BMD within a clinically well-characterized cohort with controlled confounders.

Animal Experiment Data: This part aimed for proof-of-concept. Using the three measured core inflammatory cytokines (IL-1β, IL-6, TNF-α) as input features, a classification model was built to distinguish between the Sham group (simulating normal state) and the OVX group (simulating severe osteoporotic state). This analysis sought to computationally validate whether limited inflammatory indicators alone are sufficient to accurately identify pathological bone states, thereby providing strong experimental support for the specificity of inflammatory biomarkers.

#### 2.5.2 Machine learning methodology

Data Preprocessing and Feature Engineering: To ensure data quality and model stability, all datasets underwent a unified preprocessing pipeline: 1) Missing Value Imputation: Median imputation for continuous variables; mode imputation for categorical variables. 2) Outlier Handling: Tukey’s fences method (using 1.5 times the interquartile range) was used to identify and handle extreme outliers in continuous variables. 3) Feature Standardization: All continuous features were standardized using Z-score normalization (mean = 0, standard deviation = 1) to eliminate scale effects. 4) Feature Selection: Random Forest algorithm was first used to calculate importance scores for all features. Final inclusion of the 22 core features was determined by combining these scores with clinical expertise and existing knowledge of bone metabolism mechanisms.

#### 2.5.3 Model training and validation

Algorithms for Classification Task: To achieve accurate classification predictions, this study utilized multiple ML algorithms, including 1) RUSBoosted Trees: An ensemble algorithm particularly suitable for handling class-imbalanced datasets. 2) Bagged Trees: Enhances robustness by reducing model variance through bootstrap aggregation. 3) Support Vector Machine (SVM): A supervised learning algorithm that utilizes various kernel functions (such as the Gaussian kernel) to identify the optimal hyperplane, applicable for non-linear data (Noble, 2006). 4) Logistic Regression: Included both efficient logistic regression (potentially optimized for large datasets) and traditional logistic regression as baseline models (Kleinbaum et al., 2002).

##### 2.5.3.1 Algorithms for Regression Task

1) Squared Exponential Gaussian Process Regression (GPR): A non-parametric regression method based on Bayesian theory, suitable for handling high-dimensional and complex data. 2) Matérn 5/2 GPR: A variant of the Gaussian process regression model that employs the Matérn 5/2 kernel function to enhance modeling capability for non-linear data (Dai et al., 2023). 3) Narrow Neural Network: A shallow neural network model adept at processing high-dimensional data, demonstrating strong performance in regression tasks (Beise et al., 2021). 4) Stepwise Linear Regression: A method that incrementally selects feature variables to construct a linear regression model (Yamashita et al., 2007).

##### 2.5.3.2 Training and Validation Strategy

All model training, hyperparameter tuning, and evaluation were performed using the Classification Learner and Regression Learner toolboxes in Matlab 2023b. Model performance was robustly assessed using 5-fold cross-validation. Hyperparameter tuning was conducted via Bayesian optimization, which automatically searched for the optimal hyperparameter combination, with the maximum number of iterations set to 100 to balance performance and computational cost.

#### 2.5.4 Model evaluation

##### 2.5.4.1 Classification Models

Evaluated using Accuracy, Precision, Recall (Sensitivity), F1-Score, AUC. Confusion matrices were used to visualize detailed classification results. Regression Models: Evaluated using Mean Squared Error (MSE), Root Mean Squared Error (RMSE), and Mean Absolute Error (MAE). Additionally, predicted-vs-actual value scatter plots and residual analysis plots were used to visually inspect model prediction bias and goodness-of-fit.

### 2.6 Statistical analysis

Experimental data are presented as Mean ± Standard Error of the Mean (SEM). Statistical analyses were performed using GraphPad Prism version 10.0. Comparisons among multiple groups were conducted using one-way ANOVA, followed by Tukey’s *post hoc* test for multiple comparisons if homogeneity of variance was assumed, or Dunnett’s T3 test if not. Comparisons between two groups were made using the unpaired Student’s t-test. A P-value <0.05 was considered statistically significant. All statistical analyses accounted for multiple comparisons using the Bonferroni method to control the Type I error rate.

## 3 Results

### 3.1 Performance Evaluation of BMD Classification Models based on the NHANES dataset

**FIGURE 2:**
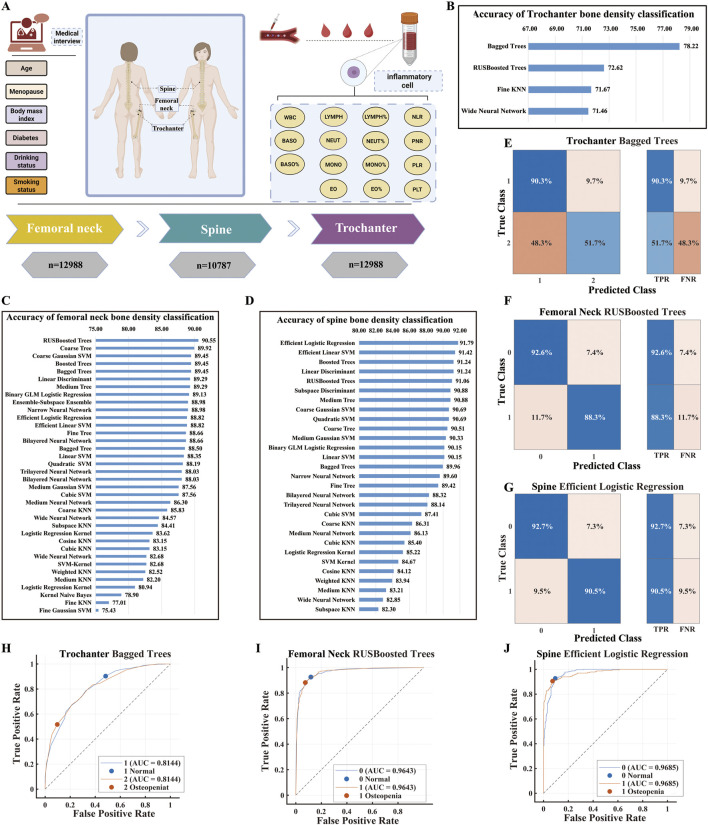
Development and Performance Evaluation of Bone Mineral Density Classification Models Using the NHANES Dataset. **(A)** Feature Overview. Shows the 22 clinical and laboratory variables used for model training, including demographics, menopausal status, BMI, medical history, lifestyle factors, blood cell counts, leukocyte subsets, and inflammatory ratios (NLR, PNR, PLR). **(B)** Trochanter BMD Classification Accuracy. Comparison of machine learning algorithms for trochanter BMD classification. Bagged Trees performed best (78.22% accuracy). **(C)** Femoral Neck BMD Classification Accuracy. Algorithm performance for femoral neck BMD classification. RUSBoosted Trees achieved the highest accuracy (90.55%). **(D)** Spinal BMD Classification Accuracy. Comparison of algorithms for spinal BMD classification. Efficient Logistic Regression showed the best performance (91.79% accuracy). **(E)** ROC Curve for Trochanter BMD. ROC curve of the optimal Bagged Trees model (AUC = 0.8144), indicating good discriminatory ability. **(F)** ROC Curve for Femoral Neck BMD. ROC curve of the best RUSBoosted Trees model (AUC = 0.9643), demonstrating excellent classification performance. **(G)** ROC Curve for Spinal BMD. ROC curve of the optimal Efficient Logistic Regression model (AUC = 0.9685), confirming strong classification capability. **(H)** Confusion Matrix for Trochanter BMD. Results for Bagged Trees model, showing high normal BMD accuracy (90.3%) but lower low BMD accuracy (51.7%) with high false negative rate (48.3%). **(I)** Confusion Matrix for Femoral Neck BMD. RUSBoosted Trees results, showing balanced accuracy for normal (92.6%) and osteopenic (88.3%) samples. **(J)** Confusion Matrix for Spinal BMD. Efficient Logistic Regression performance, with high accuracy for both normal (92.7%) and osteopenic (90.5%) samples.

This study systematically evaluated the performance of multiple machine learning algorithms in binary classification of BMD across three distinct skeletal sites using the NHANES database. As shown in Figure 2A, the analytical framework incorporated 22 feature variables encompassing demographic indicators (age, menopausal status), anthropometric parameters (BMI), clinical history (diabetes, corticosteroid use), lifestyle factors (smoking, alcohol consumption), and systemic inflammatory markers. Notably, beyond conventional white blood cell counts and differentials (including absolute counts and percentages of lymphocytes, monocytes, neutrophils), we innovatively introduced composite inflammatory indices—NLR, PLR and PNR—which provide more stable indicators of chronic inflammatory status.

Comparative analysis of algorithm performance at the femoral trochanter (Figure 2B) revealed that Bagged Trees achieved the highest classification accuracy (78.22%), followed by RUSBoosted Trees (72.62%). Fine KNN and Wide Neural Network demonstrated comparable performance (71.67% and 71.46%, respectively). The ROC curve analysis for the optimal model (Figure 2E) yielded an AUC of 0.8144, indicating moderate discriminative capability. However, examination of the confusion matrix (Figure 2H) uncovered substantial model limitations: while classification accuracy for normal BMD samples reached 90.3%, sensitivity for low BMD samples dropped sharply to 51.7%, accompanied by a false negative rate of 48.3%, revealing critical deficiencies in identifying high-risk individuals.

In contrast, substantially enhanced performance was observed at the femoral neck and lumbar spine sites. For femoral neck BMD classification (Figure 2C), RUSBoosted Trees attained the highest accuracy (90.55%). Other strong performers included Coarse Tree, Coarse Gaussian SVM, Boosted Trees, and Bagged Trees, all achieving accuracies exceeding 89%, while Fine Gaussian SVM performed poorest (75.43%). The ROC curve for this optimal model (Figure 2F) approached the upper-left corner with an AUC of 0.9643, demonstrating excellent classification efficacy. The confusion matrix (Figure 2I) confirmed balanced performance across classes, with accuracies of 92.6% for normal and 88.3% for osteopenic samples, and an FNR of 11.7% versus an FPR of 7.4%.

At the lumbar spine (Figure 2D), Efficient Logistic Regression achieved optimal performance (91.79%), closely followed by Efficient Linear SVM (91.42%) and Boosted Trees (91.24%). Overall classification accuracies ranged between 85% and 92% across algorithms, with relatively minor performance variations. Subspace KNN showed the lowest accuracy (82.30%), and most KNN variants performed below 85%, suggesting inherent limitations of KNN-based approaches for this specific classification task. ROC analysis of the top-performing model (Figure 2G) demonstrated a stable AUC of 0.9685, while the confusion matrix (Figure 2J) confirmed high and Balanced classification accuracy for both normal (92.7%) and osteopenic (90.5%) samples.

### 3.2 Error Analysis of BMD regression prediction models based on the NHANES dataset

**FIGURE 3:**
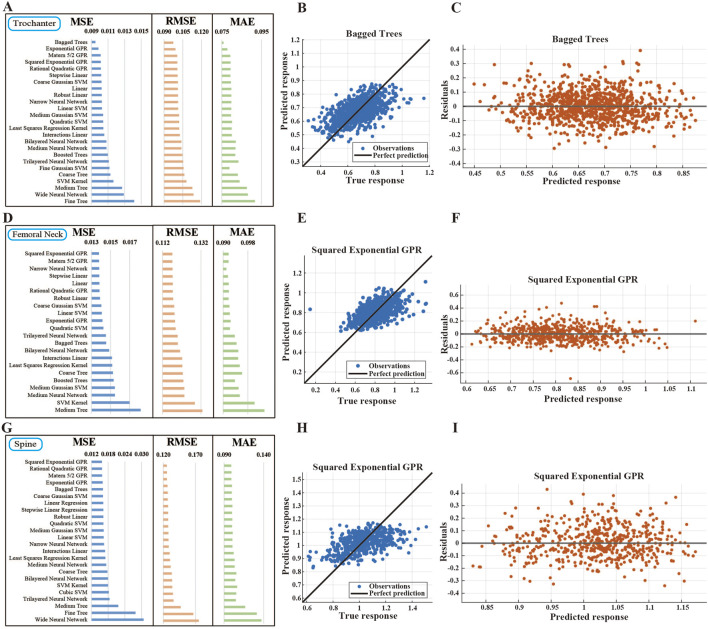
Error Analysis and Validation of BMD Regression Prediction Models Using NHANES Data **(A)** Error assessment for trochanter BMD prediction. Performance comparison of regression algorithms using MSE, RMSE, and MAE. Bagged Trees demonstrated optimal performance across all error metrics. **(B)** Predicted vs. actual values for optimal trochanter BMD model. Scatter plot of Squared Exponential GPR predictions, showing close alignment with the ideal prediction line, with minor deviations at higher values. **(C)** Residual plot for optimal trochanter BMD model. Residual distribution of Squared Exponential GPR model, showing random scatter around zero with no apparent patterns. **(D)** Error assessment for femoral neck BMD prediction. Comparison of model performance metrics. Squared Exponential GPR achieved the lowest errors across all evaluated metrics. **(E)** Predicted vs. actual values for optimal femoral neck BMD model. Scatter plot of Squared Exponential GPR predictions, demonstrating high accuracy with points closely distributed along the diagonal. **(F)** Residual plot for optimal femoral neck BMD model. Residual distribution of Squared Exponential GPR model, showing minimal spread and no systematic patterns. **(G)** Error assessment for spinal BMD prediction. Performance comparison of regression models. Squared Exponential GPR consistently showed the lowest prediction errors. **(H)** Predicted vs. actual values for optimal spinal BMD model. Scatter plot of Squared Exponential GPR predictions, showing strong linear correlation despite slight deviations at higher ranges. **(I)** Residual plot for optimal spinal BMD model. Residual distribution uniformly scattered around zero, confirming stable predictive performance across different BMD levels.

We further assessed machine learning model performance in continuous BMD prediction through comprehensive regression analysis. For femoral trochanter BMD prediction (Figure 3A), Bagged Trees consistently outperformed other models across all error metrics, achieving the lowest MSE (≈0.009), RMSE (≈0.09), and MAE values. In comparison, Fine Tree exhibited the highest MSE (≈0.015), indicating substantially larger prediction errors. The scatter plot of predicted versus actual values for Squared Exponential GPR (Figure 3B) showed most data points closely aligned with the diagonal, though some deviation was observed in higher prediction ranges. Residual analysis (Figure 3C) revealed uniform distribution around zero without systematic patterns, confirming model stability.

For femoral neck BMD prediction (Figure 3D), Squared Exponential GPR demonstrated superior performance with the lowest MSE (≈0.013), RMSE, and MAE values, while Medium Tree showed the poorest performance (MSE ≈ 0.018). The predicted-actual scatter plot (Figure 3E) displayed dense clustering along the diagonal, indicating strong agreement between predictions and measurements. Residual analysis (Figure 3F) further validated model accuracy, showing randomly distributed errors within a narrow range.

Similarly, for lumbar spine BMD prediction (Figure 3G), Squared Exponential GPR again achieved optimal performance with minimal MSE (≈0.015), RMSE (≈0.12), and MAE values. In contrast, Wide Neural Network and Fine Tree produced the highest errors (MSE ≈ 0.031 and 0.027, respectively). The prediction scatter plot (Figure 3H) indicated generally close alignment with the ideal fit line, despite minor deviations at higher BMD values. Uniform residual distribution without systematic bias (Figure 3I) further confirmed model robustness across the full BMD spectrum.

### 3.3 Validation of femoral BMD prediction model using the CPW-BMI dataset

**FIGURE 4:**
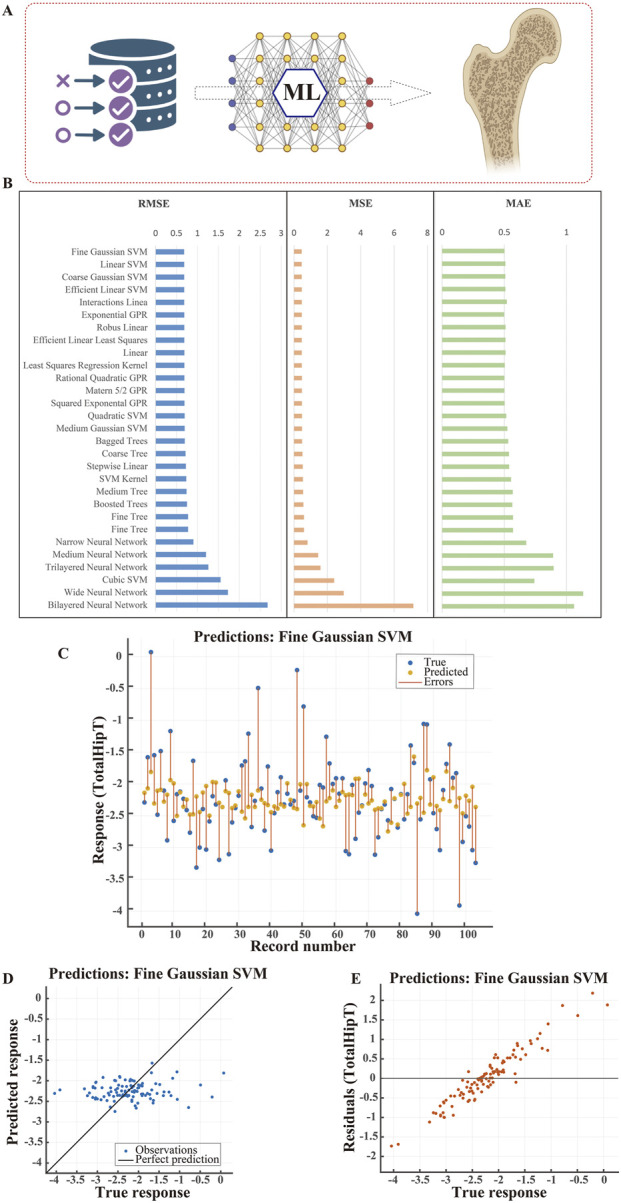
Performance Comparison and Visualization of Machine Learning Models for Femoral Bone Density Prediction (CPW-BMI Dataset) **(A)** Study workflow. Schematic overview of developing machine learning (ML) models utilizing clinical and biochemical indicators from the CPW-BMI dataset to predict femoral BMD T-scores. **(B)** Model performance comparison. Evaluation of various ML models using RMSE, MSE, and MAE metrics. Fine Gaussian SVM demonstrated the best predictive performance across all three error metrics. **(C)** Sample-wise prediction comparison. Comparison of true versus predicted T-scores using the Fine Gaussian SVM model. Blue dots represent true values, orange dots represent predictions, and orange lines indicate prediction errors. **(D)** Predicted vs. true value scatter plot. Scatter plot of Fine Gaussian SVM predictions against actual values, with the black diagonal line representing ideal predictions. **(E)** Residual plot. Distribution of prediction residuals versus true T-scores for the Fine Gaussian SVM model, demonstrating stable fitting performance across different bone density levels.

We externally validated the established prediction models using the Chinese Postmenopausal Women (CPW-BMI) cohort. The analysis pipeline (Figure 4A) involved extracting predictive features from multi-dimensional clinical and biochemical data, with special emphasis on inflammatory cytokines (IL-6, IL-1β, TNF-α).

Performance evaluation of eight major machine learning algorithms (Figure 4B) identified the Fine Gaussian SVM model as superior, demonstrating the lowest prediction bias (RMSE = 0.681, MSE = 0.464, MAE = 0.501). Multi-level visualization confirmed its performance: a sample-by-sample prediction comparison (Figure 4C) showed high agreement between model-predicted and DXA-measured values. The correlation scatter plot (Figure 4D) confirmed strong accuracy, with data points tightly clustered around the black diagonal. Residual analysis (Figure 4E) indicated uniformly random distribution of prediction residuals across BMD levels. These statistical properties collectively demonstrate the model’s stable predictive performance and robustness.

### 3.4 Inflammatory factor differential analysis and machine learning prediction of femoral BMD in the clinical validation cohort

**FIGURE 5:**
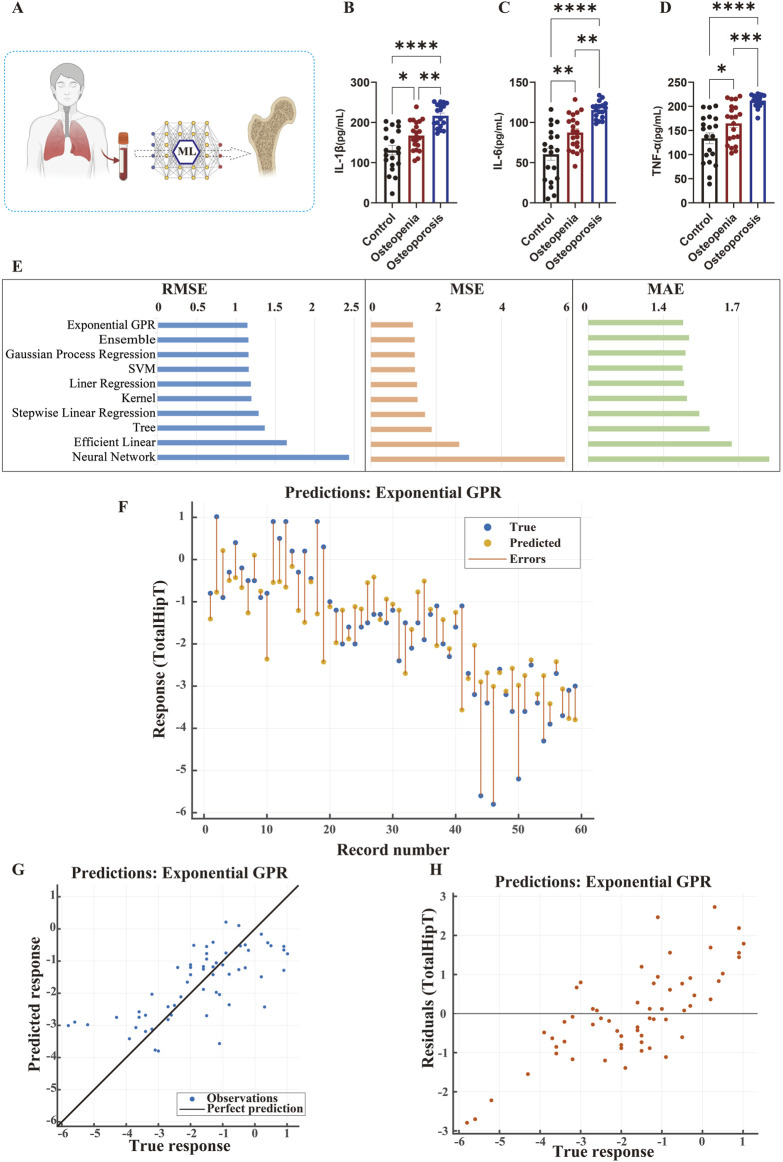
Inflammatory Cytokine Levels and Machine Learning-Based Femoral Bone Density Prediction in the Clinical Validation Cohort **(A)** Study workflow. Schematic diagram showing participant screening, serum inflammatory cytokine measurement, bone density assessment, and development of machine learning (ML) models for predicting femoral BMD T-scores. **(B–D)** Comparison of serum inflammatory cytokine levels. IL-1β **(B)**, IL-6 **(C)**, and TNF-α **(D)** levels across different bone metabolic status groups. The osteoporosis group showed significantly higher inflammatory cytokine levels compared to the osteopenia and healthy control groups. **(E)** Model performance comparison. Evaluation of various ML models using RMSE, MSE, and MAE metrics. The Exponential Gaussian Process Regression (Exponential GPR) model demonstrated the best predictive performance. **(F)** Sample-wise prediction results. Comparison of true versus predicted T-scores using the Exponential GPR model. Blue dots indicate true values, orange dots represent predictions, and orange lines show prediction errors. **(G)** Predicted vs. true value scatter plot. Scatter plot of Exponential GPR predictions, with data points closely distributed along the black ideal prediction line, indicating high prediction accuracy. **(H)** Residual plot. Residual distribution of the Exponential GPR model across true T-score ranges, demonstrating uniform distribution and confirming model stability and generalization capability.

Within the clinical validation cohort, we systematically investigated the association between inflammatory factors and BMD. The study enrolled 60 naturally postmenopausal women, rigorously matched into Osteoporosis (n = 20), Osteopenia (n = 20), and Healthy Control (n = 20) groups (Figure 5A).

ELISA revealed significant gradient differences in serum inflammatory factor levels among the three groups (Figures 5B–D). Specifically, serum IL-1β concentration was significantly higher in the osteoporosis group. Similar trends were observed for IL-6 and TNF-α. One-way ANOVA confirmed statistically significant intergroup differences, with trend tests indicating a clear dose-response relationship between inflammatory factor levels and osteoporosis severity.

In predictive model construction (Figure 5E), the Exponential Gaussian Process Regression (GPR) model performed best (RMSE = 1.135, MSE = 1.289, MAE = 0.883). Sample-by-sample predictions (Figure 5F) demonstrated high accuracy with minimal deviation from the true values. The prediction scatter plot (Figure 5G) further confirmed a strong agreement between the predicted and actual measurements, as data points were densely distributed along the ideal prediction line. Residual analysis (Figure 5H) demonstrated uniformly random distribution, confirming consistent predictive performance across individuals with different BMD levels.

### 3.5 Validation in an OVX-Induced mouse osteoporosis model and low BMD identification based on inflammatory factors

**FIGURE 6:**
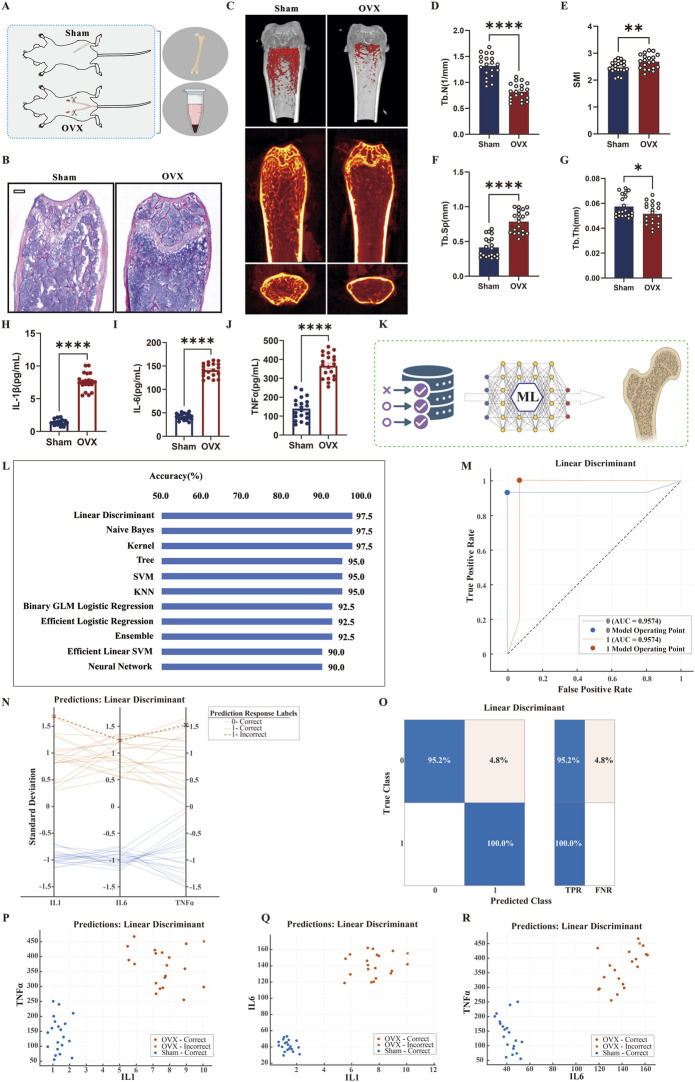
Validation of the Ovariectomy (OVX)-Induced Osteoporosis Mouse Model and Machine Learning-Based Identification of Low Bone Density **(A)** Experimental design. Schematic of the study using 12-week-old female C57BL/6J mice randomly assigned to sham-operated (Sham, n = 20) or ovariectomized (OVX, n = 20) groups to establish the postmenopausal osteoporosis model. **(B)** H&E staining of distal femur. Histological sections showing significantly reduced trabecular number, sparse arrangement, and disrupted bone structure in the OVX group compared to Sham. Scale bar: 200 μm. **(C)** Micro-CT 3D reconstruction. Images demonstrating trabecular bone loss and cortical thinning in the OVX group (n = 20). **(D–G)** Quantitative Micro-CT parameters. Compared to Sham, OVX mice showed significantly decreased trabecular number (Tb.N, **(D)**, increased structural model index (SMI, **(E)**, elevated trabecular separation (Tb.Sp, **(F)**, and reduced trabecular thickness (Tb.Th, **(G)** (n = 20). **(H–J)** Inflammatory cytokine ELISA results. Plasma levels of IL-1β **(H)**, IL-6 **(I)**, and TNF-α **(J)** were significantly elevated in the OVX group (n = 20). **(K)** Workflow and ML analysis framework. Schematic illustrating the development of machine learning models using inflammatory cytokines as features to identify low bone density status. **(L)** Classification accuracy comparison. Performance of different ML algorithms, with Linear Discriminant achieving the highest accuracy (97.5%). **(M)** ROC curve of Linear Discriminant model. The AUC value of 0.9574 indicates excellent discriminative performance. **(N)** Prediction visualization. Display of response differences between correctly and incorrectly classified samples. **(O)** Confusion matrix. Shows classification accuracy exceeding 95% for both Sham and OVX groups. **(P–R)** Feature distribution scatter plots. Two-dimensional projections based on IL-1β, IL-6, and TNF-α feature space demonstrate clear separation between Sham and OVX groups.

We utilized an animal model to explore the causal relationship between inflammation and bone metabolism. Healthy 12-week-old female C57BL/6J mice were randomly assigned to Sham (n = 20) or Ovariectomy (OVX, n = 20) groups (Figure 6A).

Histomorphometric analysis of H&E-stained sections from the distal femur (Figure 6B) revealed typical osteoporotic pathology in the OVX group, characterized by markedly reduced trabecular number, disrupted continuity, and sparse arrangement. Micro-CT 3D reconstructions (Figure 6C) visually demonstrated trabecular structural loss and cortical thinning in OVX mice. Quantitative analysis (Figures 6D–G) confirmed a significant decrease in Trabecular Number (Tb.N) and Trabecular Thickness (Tb.Th), alongside an increase in Structural Model Index (SMI) and Trabecular Separation (Tb.Sp) in the OVX group. Mechanistically, ELISA detected profoundly elevated plasma levels of inflammatory factors in OVX mice (Figures 6H–J), with TNF-α concentration increasing by 171.8% compared to the Sham group.

Using these three core inflammatory factors, we constructed a machine learning classification model (Figure 6K). Among eight different classifiers, the Linear Discriminant model achieved optimal performance with 97.5% accuracy (Figure 6L).

ROC analysis yielded an area under the curve (AUC) of 0.9574 (Figure 6M). At the cutoff value determined by the Youden index, the model achieved a sensitivity of 96.5% and a specificity of 98.2%. Visualization of model prediction response values (Figure 6N) showed nearly complete separation between groups. The confusion matrix (Figure 6O) confirmed high classification accuracy for both Sham and OVX groups. Feature space projection plots (Figures 6P–R) generated via t-SNE visualization clearly showed distinct separation between Sham and OVX groups in the high-dimensional space defined by inflammatory factors.

## 4 Discussion

This multi-level, multicenter study successfully developed and validated machine learning models for osteoporosis prediction based on inflammatory biomarkers. Our findings not only demonstrate the excellent performance of machine learning in osteoporosis risk assessment but also, from a computational medicine perspective, reveal the critical role of inflammatory markers in the pathogenesis of this complex disease.

A key observation was the notable difference in model performance across skeletal sites. At the femoral neck and lumbar spine—sites rich in trabecular bone—the RUSBoosted Trees and Efficient Logistic Regression models achieved outstanding predictive performance (AUC >0.96). In contrast, performance was more limited at the femoral trochanter, a site predominantly composed of cortical bone (AUC = 0.8144). This discrepancy holds significant biological relevance. Trabecular bone possesses a larger surface area, richer blood supply, and higher metabolic activity, fostering more intimate interactions between immune cells and bone cells within its marrow microenvironment (Fischer and Haffner-Luntzer, 2022; Tsukasaki and Takayanagi, 2019). Furthermore, the observed site-specific predictive efficacy can be explained by fundamental differences in the bone marrow microenvironment (Johnson et al., 2020). The femoral neck and lumbar spine are rich in hematopoietic red marrow, which maintains an immune-active environment with abundant macrophages and lymphocytes that interact closely with systemic inflammatory signals (Pundole et al., 2017). In contrast, the femoral trochanter is predominantly composed of adipocytic yellow marrow, which is immunologically more quiescent (Moore and Dawson, 1990). This distinction in marrow composition likely underlies why systemic inflammatory markers show superior predictive value at the primarily trabecular sites. Our results provide strong evidence from large-scale population data supporting the hypothesis that systemic inflammatory markers exhibit superior predictive efficacy at trabecular-rich sites, identifying them as primary targets for inflammatory bone loss. The superior performance of RUSBoosted Trees at the femoral neck may stem from its ensemble learning mechanism, effectively handling class imbalance common in BMD data, while the efficiency of logistic regression at the spine may reflect its capacity to capture linear patterns in high-dimensional feature spaces.

A major innovation of this study was the incorporation of composite inflammatory indices (NLR, PLR, PNR) into the predictive framework. These ratios, derivable directly from routine blood tests, offer unique systemic biological insights and significant potential for clinical translation. Mechanistically, an elevated NLR may indicate activation of Neutrophil Extracellular Traps (NETosis), a process shown to directly promote bone resorption via the release of enzymes like neutrophil elastase. PLR alterations reflect the dynamic balance between platelet activation and lymphocytic immunomodulation, and activated platelets release factors like Platelet-Derived Growth Factor (PDGF), known to indirectly influence bone metabolism (Zinellu et al., 2022; Zinellu and Mangoni, 2024). The PNR, an emerging indicator, may offer a more comprehensive reflection of the complex interplay between bone marrow hematopoietic function and systemic inflammatory status. The associations uncovered by our machine learning models not only validate known biological pathways but also provide important clues for exploring novel mechanistic links.

Our multi-tiered validation strategy—spanning population data to animal experiments—provides a complete chain of evidence supporting a causal relationship between inflammation and osteoporosis. External validation in a Chinese cohort confirmed the model’s cross-ethnic applicability and highlighted the universal nature of the association between inflammatory markers and BMD. In the prospective clinical cohort, we observed a clear dose-response relationship, with IL-1β, IL-6, and TNF-α levels showing significant graded increases across the control, osteopenia, and osteoporosis groups. This finding provides compelling clinical epidemiological evidence for a causal role of inflammation in osteoporosis pathogenesis.

Notably, the animal experiments not only recapitulated the characteristic features of postmenopausal osteoporosis but also provided direct mechanistic insights. We found that OVX mice, alongside the development of typical bone microstructural deterioration, exhibited a pro-inflammatory macrophage polarization shift and inflammasome activation within the bone marrow microenvironment. The machine learning classifier, built solely on three core inflammatory factors, achieved 97.5% accuracy (AUC = 0.9574), strongly suggesting that systemic inflammatory status represents a highly specific biomarker for osteoporosis. Different inflammatory cytokines likely impact bone metabolism via distinct signaling pathways: IL-6 primarily promotes osteoclast differentiation through trans-signaling; TNF-α synergistically enhances RANKL signaling via NF-κB and MAPK pathways; and IL-1β, one of the most potent bone resorption inducers, directly activates osteoclast precursors (Fischer and Haffner-Luntzer, 2022; Kitaura et al., 2020; Wang et al., 2019). These findings functionally link estrogen deficiency, immune system activation, and bone metabolic imbalance, offering a new theoretical framework for understanding postmenopausal osteoporosis pathogenesis.

From a translational perspective, our predictive models hold substantial clinical value. Specifically, our model could be implemented as a cost-effective, first-line screening tool in primary care settings, efficiently identifying high-risk individuals for subsequent DXA confirmation. Furthermore, it may serve as a valuable complement to established tools like FRAX, providing critical inflammatory biomarker data for patients in the intermediate-risk category. To realize this potential, future work will focus on developing a user-friendly web-based calculator and initiating large-scale prospective studies to validate its effectiveness in reducing fracture incidence within real-world clinical workflows. Their reliance on routinely available blood test parameters facilitates potential widespread adoption across healthcare institutions, aiding the establishment of early warning systems. Furthermore, the continuous prediction values enable dynamic monitoring and quantitative assessment of an individual’s bone loss rate, informing precise intervention strategies. Most importantly, the incorporation of inflammatory markers opens new avenues for prevention and treatment, suggesting that interventions targeting specific inflammatory pathways could become a pivotal future direction for osteoporosis management.

We must also acknowledge the study’s limitations. Although multiple independent datasets were used for validation, the ethnic and geographical distribution of samples remains limited, potentially affecting the model’s global generalizability. Secondly, while we focused on systemic inflammatory markers, localized inflammatory changes within the bone microenvironment and their interaction with systemic inflammation warrant further investigation (Straub, 2007; Baum and Gravallese, 2016). Regarding model interpretability, although feature importance analysis confirmed the key role of inflammatory indicators, the “black-box” nature of complex models persists. Future work will integrate advanced explainable AI techniques, such as SHAP and lime, to provide more insightful model interpretations (Vimbi et al., 2024; Ali et al., 2023; Loh et al., 2022).

Our findings, which establish a robust predictive framework for osteoporosis using inflammatory biomarkers, resonate with recent advances in adaptive machine learning for clinical diagnostics. Notably, the successful application of Few-Shot Class-Incremental Learning (FSCIL) in dynamic medical scenarios underscores the broader potential of sophisticated ML models to handle data-scarce and evolving clinical tasks (Zhang et al., 2025a; Zhang et al., 2025b; Zhang et al., 2024). Building on this foundation, we propose several directions for future research: large-scale prospective cohort studies to validate the value of inflammation-based risk models in guiding clinical interventions; utilization of single-cell sequencing and spatial transcriptomics to delineate the intricate interaction networks between immune and bone cells within the marrow niche, elucidating the precise molecular mechanisms; and interventional studies to evaluate the efficacy of therapeutics targeting specific inflammatory pathways. Furthermore, exploring the integration of our framework with an FSCIL paradigm could be a promising step towards building a truly forward-compatible system capable of seamlessly incorporating new diagnostic criteria or patient cohorts without catastrophic forgetting (Zhang et al., 2025c).

In conclusion, through an innovative design and multi-level validation strategy, this study has established a machine learning prediction framework for osteoporosis based on inflammatory biomarkers. It not only provides an effective tool for early screening but also offers novel insights into the disease’s pathogenesis. These findings are poised to advance osteoporosis management towards precision and personalization, ultimately contributing to reducing the global burden of osteoporotic fractures. We anticipate that inflammation-based risk assessment and intervention strategies will play an increasingly vital role in the comprehensive management of osteoporosis, representing a significant advancement for computational medicine in the field of skeletal health.

## Data Availability

The original contributions presented in the study are included in the article/Supplementary Material, further inquiries can be directed to the corresponding authors.
