# Supplementary material for: The critical role of inflammation in osteoporosis prediction unveiled by a machine learning framework integrating multi-source data
Source: Front Physiol. 2025 Dec 10;16:1729997. doi: 10.3389/fphys.2025.1729997 (PMC12727551; doi:10.3389/fphys.2025.1729997)
Supplement: Supplementary file 1 [file DataSheet1.pdf]

Supplementary Tables S1

Baseline Characteristics of the CPW-BMI Cohort.

	Control Group n=(5)	Osteopenia Group n=(4)	Osteoporosis Group n=(33)
Age (years), mean±SD	71.5±4.6	71.0±4.3	70.9±4.0
BMI (kg/m²), mean±SD	22.3±1.3	23.7±2.1	24.3±2.0
Femoral Neck BMD Tscore, mean±SD	-2.133±0.73	-2.220±0.68	-2.262±0.65

BMD, bone mineral density; SD, standard deviation.

Supplementary Tables S2

Baseline Characteristics of the OP-VC Cohort.

	Control Group （n=20）	Osteopenia Group （n=20）	Osteoporosis Group （n=20）
Age (years), mean ± SD	59.2± 6.6	64.6 ± 9.6	70.2 ± 9.3
BMI (kg/m²), mean ± SD	24.1 ± 1.7	24.3 ±1.3	22.7 ± 2.0
Femoral Neck BMD Tscore, mean ± SD	-0.066 ± 0.68	-1.619 ± 0.39	-3.639± 0.99

BMD, bone mineral density; SD, standard deviation.
